# In Centralized Health Systems, Much Is Left Out When Analyses of Local HiAP Strategies Are Limited to Public Administration: Comment on "A Realist Explanatory Case Study Investigating How Common Goals, Leadership, and Committed Staff Facilitate Health in All Policies Implementation in the Municipality of Kuopio, Finland"

**DOI:** 10.34172/ijhpm.2022.7646

**Published:** 2023-01-01

**Authors:** Eric Breton, Yann Le Bodo

**Affiliations:** Department SHS and Arènes Research Unit (UMR CNRS 6051: team INSERM U1309), EHESP French National School of Public Health, Rennes, France.

**Keywords:** HiAP, System Theory, Governance, Community Health, Health Inequalities, France

## Abstract

We argue that the lessons drawn by Guglielmin and colleagues, from the Health in All Policies (HiAP) approach in the municipality of Kuopio, are of limited use to centralised health systems. There is a need for research more attuned to the circumstances of local governments that have little power over the provision of health programmes; yet can address a range of determinants of population health. In these cases, adopting a state-centric perspective may fail to capture the role of other actors such as non-governmental organizations (NGOs) and local branches of state agencies. Evidence from France shows that centralised health systems can foster HiAP locally through political commitment and dedicated coordination staff whose role is to mobilise and support NGOs, inhabitants, and other local branches of regional and central governments. We highlight, as three important challenges, the issue of legitimacy, funding and positioning of the HiAP instrument in the local government structure.

## Introduction

 In their paper, Guglielmin et al^1^ provide a convincing analysis of the implementation of the Health in All Policies (HiAP) governance in the municipality of Kuopio (Finland). Much credit ought to be given to their contribution as there are too few theory-based analyses in this field that offer transferable knowledge and applicable lessons in other places. In this commentary, we argue that, albeit valuable, the Kuopio case study can do little to advance practice and policymaking in countries where health and other government responsibilities are centralised at the national level. We conclude by calling for a broader research agenda on HiAP.

###  What Can the Implementation of HiAP Bring to Population Health? 

 An HiAP perspective on improving population health is certainly essential for public health practitioners and policy-makers who believe in the powerful role that local, regional and national governments can play in improving living conditions and thus in reducing health inequities.^2^

 But the value of the HiAP approach may, at times, go well beyond incremental improvements in the way governments operate. Its full potential is best observed in tackling complex issues that current governance configurations and processes are unable to address effectively. Social inequalities (and their reproduction across generations), mental health issues, gender-based violence and adaptation to global warming are just a few examples of complex problems that not only require the mobilization of a diversity of sectors and resources within societies, but also require innovative solutions.

 While Guglielmin et al^1^ rightly remind us that part of the impetus for HiAP is rooted in a holistic perspective on the social determinants of health – and that health is indeed much more than the absence of disease – it is also worth noting that the outcome of a HiAP instrument cannot be fully understood through a traditional causal perspective. The analyst of a local policy-making process that involves a wide range of partners and who examines its various artefacts (minutes of meetings, reports, memorandum of understanding, etc) may be surprised by the innovative nature of the solutions that result from such processes. These solutions often lie at the intersection of different sectors such as housing and mental health, urban planning and physical activity and climate change and nutrition. From a system theory perspective, when new connections are established within a system of actors, they enable the mobilization of new agents and resources that can generate emergent properties (ie, new behaviours, objects, and environments that cannot be deduced from the singular properties of the people or means involved). In the complexity theory literature, a classic illustration of emergent properties is the meeting of oxygen and hydrogen to form a molecule (water) whose properties cannot be subsumed to the individual added properties of the two types of atoms. The community health literature is peppered with examples of such phenomena.^3,4^ In short, HiAP embodies causal processes that lead to transformations that cannot be fully captured through a linear Cartesian and clockwork lens on the nature of things and phenomena. In the human and natural worlds, generative causality has also to be accounted for.^5^

 Let us now look at what we can learn (or not) from the Finnish case.

###  The Limits of the Finnish Case for Drawing Lessons for Other Health Systems

 One issue left unaddressed is that of power or, more specifically, the locus of political power in decision-making.^6^ We do not believe that Finland’s presence at the forefront of the adoption/implementation of a HiAP approach is coincidental. Guglielmin et al^1^ remind us of the country’s achievements in implementing a democratic ethos, with much of the public services provided by local governments whose elected officials and civil servants enjoy great social proximity. Many people are amazed by the stories of elected representatives cycling to parliamentary sessions or doing their shopping like any normal person. In Finland, there is clearly a sense of closeness between the political elite and their constituents that is difficult to achieve in the context of centralised governance.

 This being said, Guglielmin et al have adopted an exclusively public service perspective on health policies, when a whole array of actors can also play their part in improving population health by drawing on the resources of the municipality. Again, for more centralised health systems at national level, municipalities often have little power over matters such as health and education, for example. France is one of the countries where mayors of local municipalities have little say on the provision of healthcare services and prevention programmes. In health matters, their mandate is officially limited to hygiene and sanitation. Yet, as we will see below, this does not mean that the implementation of a HiAP instrument is not feasible.^7^

 This public service perspective on HiAP also leads the authors to turn a blind eye to the influence that non-state actors (non-governmental organizations [NGOs], local businesses…) play in policy development and implementation. Yet, when vested interests hinder efforts towards a cross-sectoral municipal approach and favour the status quo, these actors can prove valuable in driving change. Beyond their intrinsic usefulness in providing tailor-made services close to the population, they can therefore be crucial in giving life to a HiAP approach over a territory.

 A key issue in HiAP research boils down to a simple question: how do you identify a HiAP scheme when looking at a set of state agencies? To their credit, Guglielmin et al provide a partial answer by framing a “successful” HiAP implementation as “policy implementation outcomes including acceptability and feasibility of implementation across parties involved, and sustainability of the HiAP implementation process.” However, they overlook an essential property of HiAP schemes, which is that they generate actions, policies and ultimately changes that are simply beyond the reach of any individual actor. In other words, they combine resources and capacities previously managed individually.

###  The Importance of Investigating Other Forms of HiAP Instrument

 France offers an interesting case of intersectoral mobilisation at the local level.^8,9^ Acknowledging the lack of coordination between the actions implemented by NGOs funded by Regional Health Agencies and different central state agencies and ministries, the 2009 Health Act^10^ opened up the possibility for Regional Health Agencies to sign agreements (ie, contracts) with local authorities setting a number of actions to improve population health. Local health contracts are not legally binding, but entail a population health need assessment that can potentially foster a new dynamic between local stakeholders. Our national survey of the local health contract,^8,9^ as well as additional analyses based on qualitative data collected in the regions of Brittany and Pays de la Loire, have highlighted some of their most interesting features.

 The first observation we can draw from our survey is the significant diversity in the topics covered by the contracts. From our sample (53 contracts out of 165 signed between January 2015 and March 2018), we can say that no two local health contracts are alike, an aspect which points to their capacity to adapt to local contexts. Their range of thematic actions goes from environmental/occupational health to substance use, nutrition, violence, housing, access to primary care, etc.

 A second feature of local health contracts is the critical role played by their coordinators. They are the hub of the HiAP apparatus. The coordinators know all the NGOs and local state agencies involved in the contract, and it is to them that these organisations turn to when they encounter problems, need support in obtaining funding or partners, or even just need a meeting room. We also found coordinators linking up NGOs with technical experts from different municipal services. They also facilitate the flow of information and ideas and are often able to quickly identify emerging issues in population groups where action is needed.

 This brings us to the third feature of the local health contracts: their ability to generate intersectoral collaboration. In analysing the actions listed in the contracts, we found many examples of solutions that required partners to work across silos. In one example, partners from the nutrition, land use and community sectors joined forces to deliver a school-based intervention to improve young people’s knowledge of healthy diets, while simultaneously creating space for an organic waste management site. In our view, and as mentioned above, such examples are clear evidence that HiAP governance is being successfully implemented.

 Yet, as much as local health contracts in France can compensate, to a certain extent, for the lack of linkages between a centralised health system and the local fabric, these contracts face many challenges that can impair their capacity to stand as HiAP instruments. As we have observed, the coordinators largely embody what local health contracts are about. The functions of coordinators and the conditions under which they are performed naturally bring us to the sociological work of Jeannot and Goodchild on local partnerships.^11^ Based on Jeannot’s earlier work,^12^ these authors identify what they call fuzzy jobs^11^ which share three main characteristics: (1) an ambiguous position in the organization; (2) a very general job description; and (3) a more precarious employment contract than that of the average public sector worker.

 These three characteristics resonate well with the multiple accounts we collected from coordinators who were recruited solely for the duration of the local health contract. These positions typically attract young professionals, a situation that explains the high staff turnover regularly reported by Regional Health Agencies. An important condition for the success of local health contracts is the involvement of elected officials. The mayor or his/her deputy in charge of health or social matters legitimises the approach and thus facilitates the mobilisation of local partners. They also determine the coordinator’s position in the local government’s organisational set-up, making him/her more or less visible and integrated into the structure. What is at stake here, in the positioning of the coordinator, is not only the institutional legitimacy that it entails, but also whether the coordinators can see themselves as fully integrated in the state apparatus or just another element that can be easily discarded. Although we lack sufficient quantitative data to support our claim, our interviews showed that the lack of effective integration of coordinators weakens the capacity of local health contracts to generate and maintain changes.

 In regard to their job description, when asked about their work, local health contract coordinators typically struggled to describe their role. What is even more remarkable is that they often undervalued their capacity to mobilise and connect with professionals from different sectors. This may suggest that the skills associated with intersectoral/HiAP approaches have yet to achieve recognition and enter mainstream training programmes.

 Finally, another element that weakens the HiAP instrument is the fact that, apart from the coordinator’s salary, the health contracts have no secured funding (an issue highlighted by Guglielmin et al^1^). Coordinators therefore invest a significant amount of their time in helping local partners to apply for grants to fund the actions listed in the contract. The experience of the current pandemic seems to have further demonstrated the importance of having HiAP instruments such as local health contracts, supported by substantial and stable funding. The funding provides some flexibility, making it easier for the local network of stakeholders to initiate and implement solutions to previously unforeseen challenges.

## Conclusion

 The study of exemplary cases such as Kuopio is undeniably essential for generating new insights. Undoubtedly, Guglielmin and colleagues’ finding on the fact that a common language among partners does not seem to be a requirement for collaboration goes against what is commonly taken for granted.^8^ However, there are valuable lessons to be drawn from cases nested within more centralised systems of governance, where most of the levers of power to alter resource allocation and change practice and policy are not locally based. In these cases, we may need to adopt a less state-centric approach to gain a clearer perspective on the alternatives in implementing HiAP. And however elusive the essence of a HiAP instrument may be, it is basically an evolving network (or a network of networks) bringing together a range of state agencies, resources and professionals working within the fabric of the broader society.

 Research and efforts towards the implementation of HiAP strategies are essential to improving population health. As public health researchers and practitioners, we are frequently reminded of how weak public health institutions are in improving the very living conditions that contribute to health inequities.^2^ The power we do have is the one we can mobilise through partnerships with other, much more powerful sectors. In other words, ours is borrowed power.

## Acknowledgements

 Authors want to thank the following members of the CLoterreS research consortium for their contribution in the conduct of the project: Yann Bourgueil, Dieinaba Diallo, Herve Hudebine, Cyrille Harpet, Françoise Jabot, and Candan Kendir.

## Ethical issues

 The CLoterreS project was reviewed by the institutional review Board of the French Institute of Medical Research and Health (INSERM) (opinion number: 18-495).

## Competing interests

 Authors declare that they have no competing interests.

## Authors’ contributions

 EB wrote the first and second version of the manuscript. YLB commented and brought significant improvements to the 2 versions.

## Funding

 This work was supported by the French Public Health Research Institute (IReSP: BRETON-AAP16-PREV-16).
